# Advances in Fungal Natural Products: Insights into Bioactivity and Therapeutic Potential

**DOI:** 10.3390/ph18091313

**Published:** 2025-09-01

**Authors:** Katarzyna Kała

**Affiliations:** Department of Medicinal Plant and Mushroom Biotechnology, Faculty of Pharmacy, Jagiellonian University Medical College, 9 Medyczna, 30-688 Kraków, Poland; k.kala@uj.edu.pl

Medicinal fungi represent a unique and underexploited reservoir of structurally diverse natural products with profound biological activities. These organisms produce a variety of secondary metabolites, including polysaccharides, terpenoids, phenolic compounds, alkaloids, proteins, and steroidal derivatives, which exhibit immunomodulatory, anti-inflammatory, antioxidant, antimicrobial, anticancer, and neuroprotective effects [1,2,3].

Advances in chemical biology, metabolomics, and pharmacology over the past decades have enabled more precise characterization of these compounds, facilitating their standardization, quality control, and potential integration into modern therapeutic strategies, such as adjuvant therapies, nutraceuticals, and functional foods [4,5,6]. Recent research has focused on understanding the molecular mechanisms underlying their bioactivity, particularly interactions with the immune system, cellular signaling pathways, and microbiota composition [7,8]. Biotechnological approaches, including fermentation and biotransformation, are increasingly employed to optimize the production of specific metabolites, while studies on synergistic effects with conventional drugs expand their potential applications in disease management [9].

Despite the longstanding interest in fungal metabolites, critical knowledge gaps remain regarding their precise mechanisms of action, optimal sources, and strategies for effective utilization in medicine. This Special Issue addresses these gaps by presenting recent advances in the isolation, characterization, and functional evaluation of bioactive fungal compounds [10,11,12,13,14,15].

The contributions to this issue provide a comprehensive overview of recent developments in the field. Coelho et al. investigated the therapeutic potential of exopolysaccharides from *Auricularia auricula* in mitigating DSS-induced colitis, demonstrating Dectin-1-mediated immunomodulation and microbiota remodeling [10]. Their findings underscore the intricate interplay between fungal polysaccharides, host immune responses, and gut microbiota, highlighting novel avenues for managing inflammatory bowel diseases [10,16,17].

Fu et al. explored the anti-inflammatory and immunomodulatory properties of *Inonotus obliquus* polysaccharides in rheumatoid arthritis using a combination of network pharmacology and experimental validation [12]. The study illustrates how integrating computational predictions with laboratory experiments can illuminate the mechanisms underlying fungal metabolite activity and guide the development of targeted therapeutics for autoimmune diseases [12,18].

Wang et al. expanded the chemical space of fungal metabolites by isolating ten novel sesquiterpenes and a new abietane-type diterpenoid from the marine fungus *Eutypella* sp., revealing potent immunosuppressive activity [15]. This work emphasizes the potential of marine-derived fungi as a reservoir of structurally unique and biologically active compounds that could serve as leads for drug development [15].

In the context of cancer therapy, Dourado et al. identified novel progesterone receptor inhibitors from fungal biotransformation metabolites, showcasing the application of bioinformatics tools in uncovering molecules with selective cytotoxicity against tumor cells [11]. Such approaches exemplify the integration of computational and experimental techniques in accelerating the discovery of bioactive fungal metabolites [11,12].

The interplay between environmental factors and secondary metabolite production is another theme highlighted in this issue. Santos et al. demonstrated that Mn(II) and Co(II) ions influence the anti-*Candida* metabolite profile of the endophytic fungus *Aspergillus* sp., revealing the importance of cultivation conditions in modulating fungal bioactivity [14].

On the other hand, Kała et al. illustrated the practical application of edible mushrooms, showing that coffee enriched with *Cordyceps militaris* and *Hericium erinaceus* fruiting bodies can serve as a convenient source of essential bioactive substances, merging dietary habits with health-promoting benefits. This work holds significant relevance for consumers, as it highlights the importance of selecting appropriate forms of supplementation to maximize health benefits [13].

Taken together, these articles highlight the vast therapeutic potential of fungal natural products while also demonstrating the diverse methodological approaches used to investigate them—from traditional biochemical assays to network pharmacology and metabolomics [10,11,12,13,14,15]. The studies collectively advance our understanding of fungal bioactive compounds, offering both mechanistic insights and translational opportunities for pharmaceutical development. Moreover, research on fungi has never progressed at such an accelerated pace, reflecting unprecedented advances in the identification, characterization, and application of bioactive fungal compounds [19,20].

Looking forward, several key areas for future research emerge. First, there is a need for standardized extraction and characterization methods to allow reproducible evaluation of bioactivity across studies. Second, integrating multi-omics approaches with in vivo validation will be critical to dissect the complex interactions between fungal metabolites, host systems, and microbiota. Third, exploring underinvestigated fungal species, including marine and endophytic fungi, may uncover novel chemical scaffolds with unique pharmacological profiles. Finally, clinical translation remains a major frontier, requiring rigorous evaluation of safety, bioavailability, and therapeutic efficacy.

In conclusion, this Special Issue underscores the promise of fungal natural products as a rich source of bioactive compounds with wide-ranging therapeutic applications. By bridging fundamental research and applied sciences, these studies provide a roadmap for future investigations that could transform fungal metabolites into clinically relevant interventions, addressing unmet medical needs while advancing our understanding of natural product biology.
